# The Institutional Library

**Published:** 1915-05-01

**Authors:** 


### THE INSTITUTIONAL LIBRARY.

### The House Surgeon's Art.

A Practical Handbook of Surgical After-treatment. By Alan H. Todd, M.S., F.R.C.S. (London: Edward Arnold. 1915. Price 4s. 6d.)

Instruction in the after-treatment of surgical cases must be looked upon as of immense importance for the medical student of to-day. Whether as house surgeon or as general practitioner he will soon find that the part he has to play after the operating surgeon has gone is one of great responsibility. To the patient the actual operation is necessarily a matter of unconcern; his impressions of the value of modern surgery will be derived from the way in which the after-treatment is carried out and the amount of discomfort he has to undergo before the ultimate result is achieved. Few will deny that the technique of careful and adequate after-treatment of surgical cases is hardly less complex than the technique of the operation itself. There are so many small details to be considered which, from the patient's point of view, are the reverse of trivial and which do indeed make a great difference to his comfort and to the progress of his convalescence. The proper appreciation of such details will greatly redound to the credit of the practitioner in charge of the case, and to him both patient and surgeon will be duly grateful. The value of an appointment as house surgeon to the newly qualified man must, in the majority of cases, lie in the practice and knowledge of after-treatment rather than in the acquirement of skill in operative procedures. There are already existing not a few handbooks of instruction in the house surgeon's art, as it may be termed, but it is safe to say that Mr. Todd's new manual will have no difficulty in maintaining itself in the front rank among such publications. The author's aim has been to provide instruction of a purely practical kind, and the fulfilment of this aim may be seen on every page. The inevitable consequence is that the author will certainly be accused of being too dogmatic, but in point of fact cavillers will only find a few sentences in this book at which they might conceivably point the finger of scorn.

Mr. Todd's position is a strong one in any case; he has himself been through the mill as dresser, house surgeon, and registrar in a first-class hospital. What he has to say about practical details may be assumed to be the outcome of actual experience, and hence we find dotted among his pages a large number of working "tips." Naturally opinions differ considerably on many of the details of after-treatment, just as surgeons have their preferences for their own little ways and instruments. Those who have experience of ways "just as good" as those in Mr. Todd's book will certainly do wisely to continue their course, but the fact remains that this handbook is one of the most helpful manuals for house surgeons that has appeared in recent years. The first six chapters are more or less general in their scope, and consequently likely to be oftenest referred to. In the chapters on the preparation for operation and on the general treatment after operation, some useful hints are given as to the use of hypnotics and analgesics. Besides special chapters relating to operations on various regions, there is a chapter devoted to bandages—a matter which is here treated somewhat briefly but not inadequately. The description of how plaster-of-Paris bandages should be applied is relegated to a subordinate position in an appendix, which might have been omitted, for these pages are not up to the standard set in the main portion of the book. Undoubtedly the first few chapters will be found the most helpful, not only for their positive statements, but also on account of the emphatic warnings they contain against practices which, although still frequently employed, are to be condemned out of hand in the light of present-day knowledge.

### On Morbid Cycles.

The Vicious Circles of Neurasthenia and their Treatment. By J. B. Hurry, M.A., M.D. Cantab. (London: J. and A. Churchill. 1915.)

This little work by a well-known investigator of vicious circles may be read with advantage by all practitioners interested in the treatment of functional nervous diseases. As Dr. Hurry points out, in few diseases does the vicious circle become so troublesome and so varied as in neurasthenia and the psycho-neuroses, and his diagrams clearly exemplify how morbid cycles arise in the various forms of these disorders. Thus nervous exhaustion leads to disordered ideas, and the inability properly to collect and control the thoughts leads to mental irritability; the latter, in its turn, increases the exhaustion of the nerve-cells, and as a consequence of this there is further disturbance of ideation, and the circle goes round again. Or, looking at the matter from another point of view, the nervous exhaustion leading to disordered ideation results in insomnia, and as nothing is more exhausting to the nervous system than sleeplessness, in this direction also a troublesome vicious circle is to be found constantly. Interesting circles that are familiar to all medical men are those in which alcoholism and the drug-habit are important links. The individual who, to tide over a neurasthenic period, takes a little alcohol, is in danger of forming a habit; and, a habit once formed, all the terrible results of chronic alcoholism soon appear, among which, of course, there is the firm desire for more stimulant which maintains the action of the circle.

The author speaks very optimistically of the relief that can be given by breaking these vicious circles, and gives various diagrams explanatory of the best methods of attacking them. Thus, where nervous exhaustion, intestinal stasis, and toxæmia are maintaining a cycle, he shows how an operation by remedying the stasis lessens the poisoning, and permits the fatigued nerve-cells to recover. But as a matter of fact those who have much to do with the treatment of the type of illness under notice know that in any case of neurasthenia, for example, so many vicious circles may be found that it is by no means as easy to bring about recovery by the breaking of one, two, or even three circles as might be supposed from a perusal of Dr. Hurry's interesting notes. The book abounds with quotations from other authors upon whose views chief reliance is sought in support of the theories advanced; however, an account of definite personal experiences is, after all, most helpful to the practical man, and in this it is noticeably lacking.

### Middle Age and Moderation.

Health for the Middle-aged. By Seymour Taylor, M.D., F.R.C.P. (Methuen. 1s. net.)

This book, one of Messrs. Methuen's Health Series, is a popular and concisely written account of the means by which health may be preserved and life prolonged for those whose youth is past by the aid of a few simple rules as to diet, clothing, recreation, etc., which are the outcome of Dr. Seymour Taylor's many years' experience amongst all classes of people. The aim of these rules is the old lesson which he thinks humanity seems less and less inclined to learn—the lesson of moderation. Dr. Seymour Taylor, for instance, draws a striking contrast in his chapter on diet between a modern business man's daily menu and the meals really necessary to preserve good health. He applies the same treatment to alcohol and tobacco, and he draws especial attention to the constant drinker who, because he is never drunk, prides himself on his moderation, but may be none the less undermining his health. He has little sympathy, however, for total abstainers, and in his dogmatic way is once or twice unfair towards them. Separate chapters are devoted to such subjects as exercise, clothing, cleanliness, recreation, sleep, and these contain information on the same popular lines. The author writes, as he says, "entirely for the layman," and his profound respect for their ignorance leads him to become somewhat platitudinous. Dr. Seymour Taylor has followed the dictum that "every man past the age of forty is either a fool or a physician," and the one fault of the book is, we think, that he is inclined occasionally to read this too literally. It should comfort the layman to be told that middle-age does not begin till forty-nine, nor end till sixty-three.

### Martindale and Westcott.

The Extra Pharmacopœia of Martindale and Westcott. Revised by W. H. Martindale, Ph.D., F.C.S., and W. Wynn Westcott, M.B., D.P.H. Sixteenth Edition. In Two Volumes. (London: A. K. Lewis. 1915. Vol. I., price 14s. Vol. II., price 7s.)

"Looking it up in Martindale and Westcott" has for years been a profitable occupation when any information has been desired on pharmaceutical or therapeutical points. Compared with some of the earlier editions the present volumes have assumed an almost encyclopædic character as a reference work on the progress made in the sciences concerned, especially in the varied offshoots and bypaths of these domains. As examples of compactness these volumes would be hard to rival, and without a careful perusal it is difficult to realise the vast amount of information which is contained in these two almost elegant pocket-books. To summarise even the scope of this work is impossible; its practical utility needs no advocate. The frequency with which members of the medical and pharmaceutical professions are wont to appeal to its pages is sufficient evidence of their appreciation of the immense amount of labour entailed in keeping the volumes up to date. In Volume II. there will be found a section which is of no little general interest at the present time. We refer to those pages which provide highly interesting information about proprietary medicines, and in which are given certain details of their composition. A more widespread knowledge of the constituents of some of these patent medicines is likely to be a potent factor in decreasing their sale, particularly in the more flagrant cases. Already the provision of qualified medical advice for the fourteen million persons who come under the National Insurance Act is having an inhibitory effect on this trade. In this volume a useful digest is given of the evidence taken by the Select Committee appointed to inquire into the conditions prevailing in the United Kingdom regarding the sale of patent and proprietary medicines.

Of the new features mention must be made of the brief references, alphabetically arranged in the preface, to many of the latest suggestions in the domain of therapeutics. Accepted ideas are in some instances directly contradicted, but much valuable information is given in a concentrated form with the results of independent investigation. Among the sections of great practical interest will be numbered that dealing with tuberculin therapy. This now very complex and much discussed form of treatment is dealt with in nearly all its modifications in a most concise and helpful manner, the lactic acid tuberculin of the Deycke-Much programme being the most important omission. The arguments for and against tuberculin are excellently summarised. It must suffice to add that this valuable compendium contains much practical information on the therapy of vaccines and antitoxins, on radiology, on the metallic colloids, and other modern agents. A list of the European spas and notes on the properties of their waters is perhaps the only section which may be described as being of limited utility at the present time. It is hardly necessary to mention that this edition of "The Extra Pharmacopœia" contains full information of the changes which have been made in the new edition of the British Pharmacopœia.

### Christian Science.

A Plea for the Thorough and Unbiassed Investigation of Christian Science. By Charles Herman Lea. (J. M. Dent and Sons, Ltd. Price 1s. net. Second and Revised Edition.)

Italics and black type are no doubt intended to make things easier for the reader, but they can be overdone, and in the volume before us they are used with a recklessness which makes what is already a highly polemical book very unreadable. It is not surprising, therefore, that the writer is as dogmatic as his italics and black letters are frequent, and such statements as "on every point medical practice is in absolute opposition to Christian Science practice" are placed side by side with the quaint argument that Christian Science practitioners should charge fees for treatment, because the payment of a fee shows that the patient takes the matter seriously. The author's statement is much more "a challenge to critics" than a plea for unbiassed investigation, and his book suffers in cogency, style, and reasoning in consequence of it.

